# Hemispheric asymmetry of [^11^C](*R)*PK11195 binding to translocator protein 18 kDa (TSPO) in normal brain

**DOI:** 10.1177/0271678X251348790

**Published:** 2025-06-19

**Authors:** Bandar Q Alfaifi, Jouni Tuisku, Markus Matilainen, Jose Anton-Rodriguez, Daniel Lewis, Alan Jackson, David J Coope, Laura Airas, Bill Deakin, Karl Herholz, Alexander Gerhard, Rainer Hinz

**Affiliations:** 1 Wolfson Molecular Imaging Centre, 5292 The University of Manchester, Manchester, UK; 2 Department of Clinical Radiological Sciences, College of Applied Medical Sciences, Jouf University, Sakaka, Saudi Arabia; 3 Geoffrey Jefferson Brain Research Centre, University of Manchester, Manchester, UK; 4 Turku PET Centre, 8058 University of Turku, Turku, Finland; 5 Turku PET Centre, Turku University Hospital, Turku, Finland; 6 Department of Nuclear Medicine, Center for Translational Neuro- and Behavioral Sciences, University Medicine Essen, Essen, Germany; 7 Department of Geriatric Medicine, Klinikum Hochsauerland, Arnsberg, Germany

**Keywords:** Asymmetry, brain, normal, TSPO, [^11^C](*R*)PK11195

## Abstract

Many positron emission tomography (PET) imaging studies in health and disease of the translocator protein 18 kDa (TSPO) using different radioligands have been published, however, only few separately reported left and right regions of interest in the brain. Thus, TSPO binding in healthy brains using [^11^C](*R*)PK11195 datasets of 76 participants from two PET sites was assessed for symmetry. Structural MRI scans were used for brain segmentation and to define six regions of interest (thalamus, putamen, temporal, frontal, parietal, and occipital cortex) with a probabilistic brain atlas. The simplified reference tissue model with bilateral grey matter cerebellar reference tissue input function was used to estimate distribution volume ratios (DVR). On a global level, the right hemisphere had higher DVR than the left side (p < 0.001, Cohen’s *d = *1.14 for grey matter and 1.17 for grey matter and white matter regions of interest). There were statistically significantly greater DVRs in all right regions with p < 0.001 except in the occipital cortex (p = 0.012, Cohen’s *d = *0.29). This asymmetry was independent of age, sex, and handedness evaluated using a linear mixed-effects model. These results demonstrate that [^11^C](*R*)PK11195 has an asymmetric binding distribution in the human brain, which needs to be accounted for in clinical studies.

## Introduction

The translocator protein 18 kDa (TSPO) is a nuclear encoded mitochondrial protein that is abundant in peripheral organs (particularly adrenal glands and kidneys) and hematogenous cells. In normal conditions, TSPO expression is low throughout the brain.^1,2^ In diseases of the central nervous system (CNS), high levels of TSPO have been observed in infiltrating blood-borne cells and in activated microglia.^3^ TSPO expression increases after neuronal damage from inflammatory, ischemic, degenerative, and neoplastic diseases.^4^ Microglia are activated not only in the surroundings of focal lesions but also in the distant, anterograde, and retrograde projection areas of the lesioned neural pathway.^5^

Few studies have demonstrated hemispheric asymmetries of immunological brain function.^6^ For instance, higher production of interleukines IL-1β and IL-6 in the right than in the left hemisphere have been reported in mice.^7^ In humans, resections in the language-dominant hemisphere due to epilepsy reduced lymphocytes, total T cells, and helper T cells in the blood, while resections in the non-dominant hemisphere had the opposite effect.^8^ However, as yet, there are no studies directly demonstrating asymmetry of immunological cell function in the human brain.

Positron Emission Tomography (PET) has been used to image TSPO *in vivo* with [^11^C](*R*)PK11195, a ligand selectively binding to TSPO, since the 1980ies. TSPO PET imaging has been used in a whole range of CNS conditions, such as aging and dementia disorders,^9–12^ stroke,^13–17^ multiple sclerosis,^18–20^ psychiatric disorders,^21–24^ and brain tumors.^25–28^ In these case-control observational studies, healthy volunteers were used as the reference group to which the data obtained in the clinical study cohort were compared.

In some of these studies, for example, in patients with stroke,^13,15^ intracerebral hemorrhage,^17^ temporal lobe epilepsy^29,30^ or brain tumors, a lesion is classified by its location in the two brain hemispheres as the ipsilateral hemisphere and the contralateral hemisphere, respectively. In normal volunteers, it is not known whether there is a difference in TSPO expression between the left and right hemisphere or between the dominant and nondominant hemisphere based on the subject’s handedness and localization of language in the brain.^31^ Characterization of the normal TSPO PET expression with respect to hemispheric laterality is, therefore, necessary to ensure accurate analysis and interpretation of such clinical imaging studies.

Imaging studies in healthy individuals using PET have reported hemispheric asymmetry in cerebral glucose metabolism^32–34^ with a predominance of the right hemisphere and magnitude ranging between 2%–3%.^35^ For instance, at regional levels, Willis et al.^34^ reported right greater than left cerebral glucose metabolic rate (CMR_glc_) in the lateral frontal and temporal regions and cerebellum, whereas left was greater than right in the lingual gyrus, medial frontal gyrus, thalamus, and superior cingulate. PET and single-photon emission computed tomography (SPECT) studies have also reported hemispheric asymmetries in cerebral blood flow (CBF).^36–41^ CBF has been measured with various techniques, including [^15^O]water PET,^36,38^ ^133^xenon SPECT,^37,39,40^ and ^99m^Tc-ECD SPECT,^41^ and reported to be greater in the right hemisphere. For instance, when measured using ^99m^Tc-ECD SPECT, the right hemisphere showed significantly higher CBF than the left hemisphere (p < 0.001), with an average asymmetry index of (+1.40).^41^

Of the hundreds of clinical TSPO PET studies in the literature, for example, reviewed by Chauveau et al.,^42^ only a few investigations were found that separately reported left and right regions of interest (ROI) in the brain.^9,10,29,43–46^ Of these seven publications, only Kumar et al.^46^ specifically tested for a statistically significant difference between left and right ROIs in 15 healthy adults scanned with [^11^C](*R*)PK11195. The repeated measures analysis of variance (ANOVA) did not find a hemispheric asymmetry. Gershen et al.^29^ assessed hemispheric asymmetry as part of their clinical study using two second-generation TSPO tracers [^11^C]PBR28 (11 control participants) and [^11^C]DPA-713 (7 control participants) and reported no significant hemispheric difference in healthy controls imaged with [^11^C]PBR28, however, healthy control scanned with [^11^C]DPA-713 showed a significant difference in uptake between the left and right in fusiform gyrus.

Post-mortem TSPO binding in healthy human brains from 13 studies^1,47–57^ was reviewed by Tong et al.,^58^ the majority of which used [^3^H]PK11195. None of these studies separately reported left and right ROIs in the brain. Therefore, it was impossible to assess whether asymmetry was noted in the post-mortem human brain.

However, there are few studies assessing asymmetry in healthy adults that might help to put these clinical findings into context. An understanding of the normal TSPO distribution in healthy brains is essential if physiological variation is to be distinguished from pathology-associated changes. In this study, we investigated the regional asymmetry in the human brain using [^11^C](*R*)PK11195 PET. We combined [^11^C](*R*)PK11195 PET data acquired on the High-Resolution Research Tomograph (HRRT)^59^ from two PET centres, resulting in one of the largest TSPO PET control datasets to date.

## Material and methods

### Subjects

This study was based on the retrospective analysis of [^11^C](*R*)PK11195 data from 76 healthy participants who had been scanned at the Wolfson Molecular Imaging Centre (WMIC), University of Manchester, UK (50 healthy volunteers) and the Turku PET Centre and Turku University Hospital, Finland (26 healthy volunteers). The data from Manchester and Turku were from several previously published studies.^19,22–25,60^ The original publications provide detailed information on inclusion and exclusion criteria, as well as ethical approvals and adherence to relevant ethical standards. Written informed consent was obtained from all participants. Descriptive statistics for age, sex, and handedness are reported in Table 1.

**Table 1.** Participants’ demographics and injected radiotracer specifics.

	Manchester	Turku	Total
Participants	50	26	76
Male/Female	30/20	8/18	38/38
Median Age (IQR) in years	40 (24–50)	43 (33–46)	42 (28–49)
Handedness (Right/Left/unknown)	44/5/1	15/3/8	59/8/9
Median injected activity (IQR) in MBq	679 (546–729)	496 (483–500)	546 (494–718)
Median injected mass of stable [^11^C](*R*)PK11195 (IQR) in µg	1.38 (0.95–2.26)	1.40 (0.80–1.90)	1.40 (0.95–2.18)

IQR: interquartile range; MBq: megabecquerel; µg: microgram.

### Structural MRI

Structural T1-weighted magnetic resonance imaging (MRI) was performed with 1.5 Tesla or 3 Tesla Philips Achieva scanner in Manchester and 1.5 Tesla Philips Gyroscan Intera Nova Dual scanner or 1.5 Tesla Ingenuity TF PET/MR scanner (Philips, Best, the Netherlands) in Turku. Structural T1-weighted MRI was utilized to exclude participants with structural abnormality and for delineation of ROIs for PET image analysis.

### [^11^C](R)PK11195 PET imaging

The [^11^C](*R*)PK11195 synthesis was similarly prepared and previously described.^19,61^ In both sites, [^11^C](*R*)PK11195 PET scans were acquired using a High-Resolution Research Tomograph (HRRT) (CTI/Siemens Molecular Imaging, TN, USA), a dedicated human brain PET camera providing a superior intrinsic spatial resolution.^59^

A 6-minute transmission scan using ^137^Cs point source was acquired before injection of the radiotracer for subsequent attenuation and scatter correction. [^11^C](*R*)PK11195 was then injected as slow bolus. The radiotracer administration parameters are presented in Table 1. Dynamic emission data were then acquired in list mode for 60 minutes after injection. In Manchester 18 timeframes (one background frame × approximately 6 min prior to injection; 1 × 15 sec; 1 × 5 sec; 1 × 10 sec; 1 × 30 sec; 4 × 1 min; 7 × 5 min; 2 × 10 min) and in Turku 17 timeframes (2 × 15 sec, 3 × 30 sec, 3 × 1 min, 7 × 5 min, and 2 × 10 min) were created.

The dynamic PET images were reconstructed with an iterative ordinary Poisson Ordered Subset Expectation Maximization (OP-OSEM) 3D algorithm using 12 iterations and 16 subsets^62^ at Manchester and 8 iterations and 16 subsets at Turku, generating images with an isotropic voxel size of 1.22 × 1.22 × 1.22 mm^3^. Corrections for detector normalization, dead time, attenuation, random and scattered coincidences were incorporated. The dynamic PET images were smoothed with a 3D Gaussian filter with a full width at half-maximum (FWHM) to increase signal to noise following image reconstruction.

### [^11^C](R)PK11195 PET analysis

Manchester imaging data consisting of the reconstructed [^11^C](*R*)PK11195 dynamic HRRT images and the structural T1-weighted MRI for each subject were transferred to Turku PET centre, where the locally acquired images were pre-processed in a similar manner. The dynamic [^11^C](*R*)PK11195 emission image (2 mm FWHM presmoothed) was used to create a summed [^11^C](*R*)PK11195 PET image, which then was co-registered to the structural T1-weighted MRI using FMRIB’s Linear Image Registration Tool (FLIRT) within the FMRIB Software Library (FSL).^63^ The inverse parameters of this affine transformation were then applied to the structural T1-weighted MRI to co-register and resliced the MRI to the summed PET image.

The co-registered T1-weighted MRI was then segmented into grey matter and white matter (WM) probability maps using a unified segmentation method^64^ in SPM12 software (Wellcome Trust Centre for Neuroimaging, London, UK) running in MATLAB (The Mathworks, Natick, MA). The inverse parameters from the non-affine transformation were subsequently used to warp the probabilistic brain Hammers atlas,^65^ which consists of 83 ROIs, from template space into individual PET spaces. These ROIs correspond to regions in the Hammers atlas were used as the basis for both global and regional analyses.

The regions of interest defined by the warped probabilistic atlas were then masked by brain contour in order to minimize the spill over effect from intense [^11^C](*R*)PK11195 signal present in soft tissue outside the brain. The SPM generated grey matter probability maps were binarized by thresholding at 0.5.^25,26^

For global analysis, we created left and right cortices defined as grey matter only by combining grey matter regions from the thresholded grey matter segmented image. A left and right whole brain region was also created by combining regions from the unsegmented brain regions and defined as grey and white matter (Brain; GMWM). At the regional level, regions from the Hammers atlas were grouped into combined ROIs. The segmented grey matter atlas was used to combine small cortical regions into eight left/right cortices ROIs, including temporal, frontal, occipital, and parietal. Four subcortical regions were also assessed, including left/right thalamus and putamen, defined as whole unsegmented regions (grey and white matter).

Dynamic [^11^C](*R*)PK11195 images (presmoothed with 3D Gaussian 4 mm FWHM) were used to create [^11^C](*R*)PK11195 binding potential (BP_ND_) parametric images, representing the ratio of specifically bound radiotracer over the non-displaceable radiotracer in tissue at equilibrium, using the simplified reference tissue model (SRTM)^66,67^ and the bilateral cerebellar grey matter ‘pseudoreference’ region time-activity curve (TAC) as reference tissue input function. The primary outcome measures were the distribution volume ratio (DVR = BP_ND_ + 1) and tracer delivery ratio (R_1_ =  K_1_/K_1_′, K_1_ where K_1_′ represent the rate constant for transfer from arterial plasma to tissue in the target region and in the reference region, respectively^68^) Regional values were read out by applying the atlas ROIs to the parametric images.

The [^11^C](*R*)PK11195 standardized uptake value (SUV_40-60_) for cerebellar grey matter was calculated by averaging the radioactivity concentration from frames spanning 40–60 minutes in the regional TAC and normalized by the injected radioactivity and body weight, as previously reported.^69^

We calculated the [^11^C](*R*)PK11195 DVR hemispheric asymmetry index as the difference between left and right divided by the sum of both, as previously described.^70^ We also calculated the relative difference between left and right as a percentage (%), defined as the absolute difference between left and right divided by the mean of both. Thus, a positive sign for both AI and relative difference indicates that the right DVR is greater than the left value. The absolute [^11^C](*R*)PK11195 DVR difference (DVR_right_-DVR_left_) was assessed to show how differences are distributed. Finally, to provide a measure of effect size, Cohen’s *d* was additionally calculated as the mean of paired differences divided by their standard deviation.

Hemispheric volume differences were evaluated at both the entire hemisphere (grey matter and Brain (GMWM)) and regional levels using the Wilcoxon matched-pairs signed rank test. We also calculated the volume asymmetry index, defined as (Right–Left)/(Right + Left), corresponding to the asymmetry index used for DVR. Pearson correlation analyses were then performed between the volume and DVR asymmetry indices.

### Statistical analysis

Statistical analysis was performed using GraphPad Prism version 10.1.0 for Mac (GraphPad Software, Boston, Massachusetts, USA). The normality of the age, radiotracer specifics, and regional [^11^C](*R*)PK11195 DVR were inspected with the Shapiro-Wilk test. All continuous variables are presented as median (interquartile range [IQR]).

**Figure 1.**
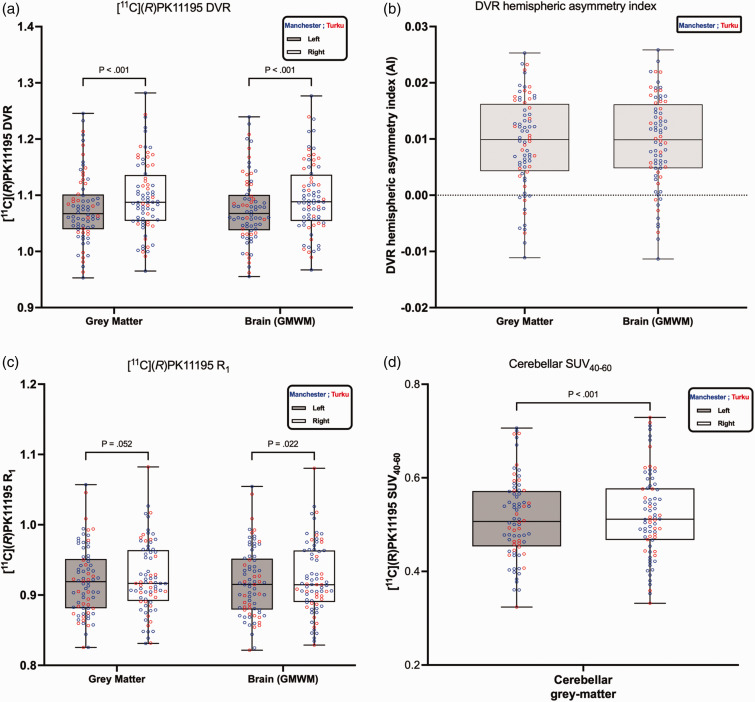
[^11^C](*R*)PK11195 distribution volume ratio (DVR), tracer delivery ratio (R_1_), and DVR hemispheric asymmetry index comparing the left and right hemispheres for grey matter and for brain tissue (GMWM = grey matter and white matter combined), as well as [^11^C](*R*)PK11195 SUV_40-60_ of cerebellar grey matter. The origin of the data is represented by the color (blue for Manchester and red for Turku). Panel (a) The boxplots represent the median DVR (interquartile range [IQR]) values; the whiskers represent min/max values, and p < 0.001 indicates the results from the Wilcoxon matched-pairs signed rank test. Panel (b) illustrates [^11^C](*R*)PK11195 DVR hemispheric asymmetry index. A positive sign indicates the right DVR is greater than the left. Panel (c) the boxplot represents the median tracer delivery ratio (R_1_) (interquartile range [IQR]) values; the whiskers represent min/max values, and p values indicate the results from the Wilcoxon matched-pairs signed rank test. Panel (d) the boxplot represents the median [^11^C](*R*)PK11195 SUV_40-60_ of cerebellar grey matter (interquartile range [IQR]) values; the whiskers represent min/max values, and p values indicate the results from the Wilcoxon matched-pairs signed rank test.

**Figure 2.**
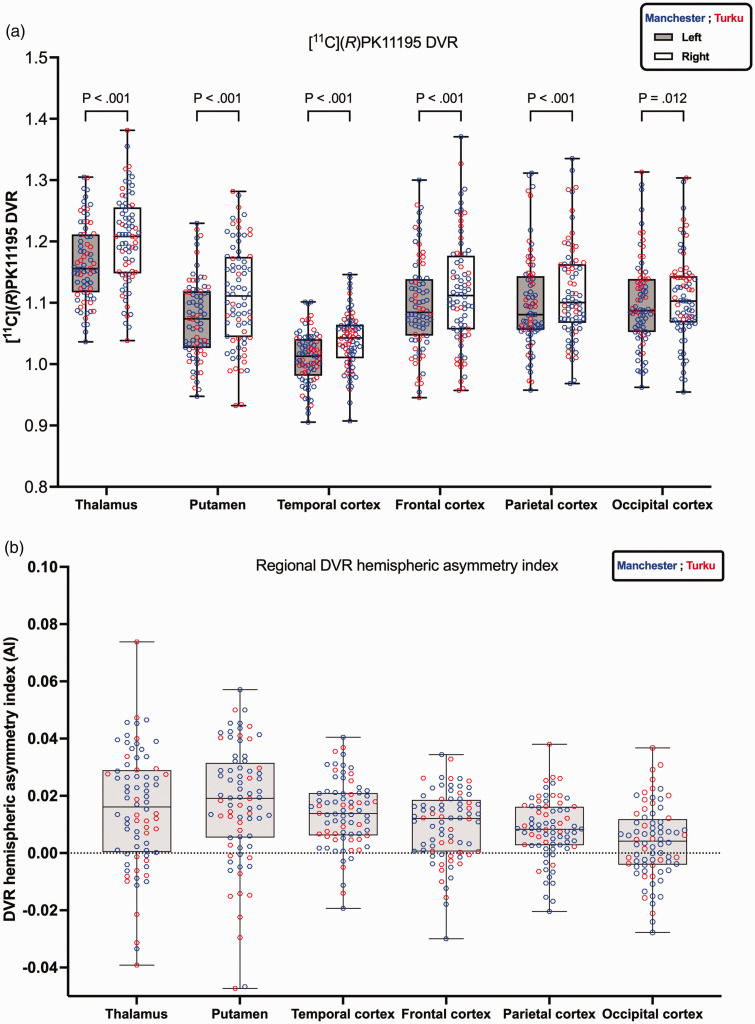
[^11^C](*R*)PK11195 distribution volume ratio (DVR) for six regions of interest comparing the left and right regions for data from Manchester (blue) and Turku (red). Panel (a) boxplots represent the median DVR values with (interquartile range [IQR]), and whiskers represent min/max values. The Wilcoxon matched-pairs signed rank test results comparing left and right regions were highly statistically significant, indicated by p < 0.001. For the occipital cortex, the difference was significant at p = 0.012. Panel (b) illustrates [^11^C](*R*)PK11195 DVR regional hemispheric asymmetry index. A positive sign indicates the right DVR is greater than the left.

The main research question of this study was to evaluate [^11^C](*R*)PK11195 regional asymmetry. For the datasets combined from both sites, [^11^C](*R*)PK11195 asymmetry (right vs left) was tested using the Wilcoxon matched-pairs signed rank test post-hoc (Figures 1(a) and 2(a)). Within-center, [^11^C](*R*)PK11195 asymmetry was also tested using the Wilcoxon matched-pairs signed rank test post-hoc, while between-center [^11^C](*R*)PK11195 DVR difference was tested using the Mann-Whitney test (Table 2). All statistical tests were carried out with multiple comparison corrections utilizing the Holm-Šídák method.

**Table 2.** [^11^C](*R*)PK11195 DVR median (IQR) values in six brain regions and in the hemisphere for grey matter and for brain tissue (grey matter and white matter combined), including the results for within-center and between-center regions evaluation.

		Manchester (N = 50)		Turku (N = 26)		Manchester vs Turku
Regions	Side	Median DVR (IQR)	Wilcoxon matched-pairs test (P value)	Median DVR (IQR)	Wilcoxon matched-pairs test (P value)	Mann-Whitney UTest (P value)
Thalamus (GMWM)	Left	1.15 (1.12–1.21)	<0.001	1.16 (1.12–1.21)	0.038	>0.05
	Right	1.21 (1.16–1.26)		1.19 (1.15–1.24)		>0.05
Putamen (GMWM)	Left	1.08 (1.03–1.12)	<0.001	1.07 (1.02–1.13)	0.041	>0.05
	Right	1.12 (1.07–1.17)		1.07 (1.01–1.21)		>0.05
Temporal Cortex	Left	1.01 (0.97–1.04)	<0.001	1.01 (0.99–1.04)	<0.001	>0.05
	Right	1.04 (1.00–1.07)		1.05 (1.02–1.06)		>0.05
Frontal Cortex	Left	1.08 (1.06–1.13)	<0.001	1.09 (1.01–1.17)	0.029	>0.05
	Right	1.11 (1.07–1.17)		1.09 (1.00–1.19)		>0.05
Occipital Cortex	Left	1.08 (1.04–1.11)	0.237	1.13 (1.09–1.18)	0.035	0.009
	Right	1.08 (1.06–1.12)		1.14 (1.12–1.16)		<0.001
Parietal Cortex	Left	1.07 (1.05–1.12)	<0.001	1.12 (1.06–1.17)	<0.001	>0.05
	Right	1.09 (1.06–1.14)		1.16 (1.09–1.19)		0.038
Grey Matter	Left	1.06 (1.04–1.09)	<0.001	1.09 (1.03–1.13)	<0.001	>0.05
	Right	1.09 (1.06–1.11)		1.11 (1.05–1.17)		>0.05
Brain (GMWM)	Left	1.06 (1.04–1.09)	<0.001	1.08 (1.03–1.13)	<0.001	>0.05
	Right	1.09 (1.06–1.11)		1.11 (1.05–1.16)		>0.05

Within center difference was tested using Wilcoxon matched-pairs signed rank test, and between center difference was tested using Mann-Whitney U test. N: number of participants; DVR: distribution volume ratio; IQR: interquartile range [being the 25th and 75th percentile]; GMWM grey matter and white matter tissues combined.

To further evaluate the [^11^C](*R*)PK11195 DVR and the individual biological factors, we used a linear mixed effects model in which subjects (repeated measures) were identified as random effects, allowing their intercepts to vary freely. Fixed effects were specified as hemispheres (left/right), age, sex, and handedness (Table 1). Age, sex, and handedness were incorporated within the model to test their effect on the [^11^C](*R*)PK11195 DVR. We included PET centres and a centre-by-hemisphere interaction term to account for any differences in image acquisition and reconstruction across different centres. Handedness information was unknown in nine participants who were then excluded from the model analysis. The results are presented as unstandardized regression effect estimates with 95% confidence intervals (CI). This analysis was conducted using “lme4” and “lmerTest” packages in R Statistical Software (version 4.2.1). We considered the ROI analyses highly dependent comparisons and set the alpha threshold to 0.05 (two-tailed).

## Results

There was no significant difference in the age between the populations from the two sites, p = 0.661 (Mann Whitney test, Table 1), confirming that the cohorts from the two scanning sites were age-matched. There was a significant difference in the injected [^11^C](*R*)PK11195 injected activities between the two sites (p < 0.001; Mann Whitney test, Table 1). There was no significant difference in the injected mass of stable [^11^C](*R*)PK11195 (p = 0.491; Mann Whitney test, Table 1).

The [^11^C](*R*)PK11195 DVR for the entire hemispheres is presented in Figure 1(a) and as the last two regions in Table 2. The visual impression in Figure 1(a) that the blue data points (Manchester) and the red data points (Turku) are well mixed was confirmed by the Mann-Whitney test between the two sites (last column in Table 2) with non-significant p-values for left and right grey matter and Brain (GMWM). Looking at the asymmetry by comparing the left with the right [^11^C](*R*)PK11195 DVR for all 76 participants using a Wilcoxon test, a statistically significant p < 0.001 was obtained, indicating that the DVR in the right hemisphere is greater than that in the left hemisphere for both tissue types: grey matter and Brain (GMWM).

The [^11^C](*R*)PK11195 DVR of six left/right brain regions (thalamus, putamen, and four cortices) are presented in Figure 2(a) and Table 2. As before, for the comparison between the hemispheres in Figure 1(a), the red and blue data points from the two sites appear well mixed when visually assessed, with the exception of the last region shown. In the occipital cortex (in particular the right occipital cortex), the lower end of the distribution is dominated by blue signs representing Manchester data. The difference between Turku and Manchester data was statistically significant only in the occipital cortex (last column in Table 2) with p = 0.009 for the left occipital cortex and p < 0.001 for the right occipital cortex and in the right parietal cortex p = 0.038 where the median DVR from Turku was greater than the median DVR from Manchester. As already found in the comparison between the left and right hemispheres, all six regions shown in Figure 2(a) had a higher median DVR on the right than the left side, with p < 0.001 from the Wilcoxon test everywhere except the occipital cortex with p = 0.012.

The [^11^C](*R*)PK11195 DVR hemispheric asymmetry index is shown in Figure 1(b) and at regional levels in Figure 2(b). At global levels, grey matter and Brain (GMWM) both showed rightward asymmetry (Figure 1(b)). Similarly, at regional levels, all regions showed rightward asymmetry (Figure 2(b)). The [^11^C](*R*)PK11195 DVR difference (DVR_right_-DVR_left_) exhibited Gaussian distribution as confirmed by Shapiro-Wilk test across both tissue types: grey matter and Brain (GMWM) (Grey matter p = 0.40; GMWM p = 0.56), as well as across brain regions. For the relative difference between the left and right hemispheres, the right hemisphere showed a higher DVR in both grey matter and whole brain (GMWM) with a difference of +1.87% (Cohen’s *d = *1.14) and +1.90% (Cohen’s *d = *1.17), respectively. At the regional level, the highest relative difference was observed in the putamen (+3.55%, Cohen’s *d = *0.84), followed by the thalamus (+3.09%, Cohen’s *d = *0.75), temporal cortex (+2.78%, Cohen’s *d = *1.19), frontal cortex (+1.96%, Cohen’s *d = *0.78), parietal cortex (+1.66%, Cohen’s *d = *0.78) and then occipital cortex (+0.76%, Cohen’s *d = *0.29).

The [^11^C](*R*)PK11195 R_1_ for the entire hemispheres across all 76 participants is presented in Figure 1(c). When assessing [^11^C](*R*)PK11195 R_1_ asymmetry, neither grey matter nor Brain (GMWM) reached a significance of 0.01 (grey matter p = 0.052; Cohen’s *d = *0.26 and Brain (GMWM) p = 0.022, Cohen’s *d = *0.30). The [^11^C](*R*)PK11195 hemispheric asymmetry of R_1_ is less significant than that observed in DVR.

The [^11^C](*R*)PK11195 SUV_40-60_ of cerebellar grey matter is presented in Figure 1(d). As highlighted in the rest of the brain regions, looking at the asymmetry by comparing the left with the right [^11^C](*R*)PK11195 SUV_40-60_ for all 76 participants using a Wilcoxon test, a statistically significant p < 0.001 was obtained, indicating that the SUV in the right grey matter cerebellum is greater than that in the left.

**Figure 3.**
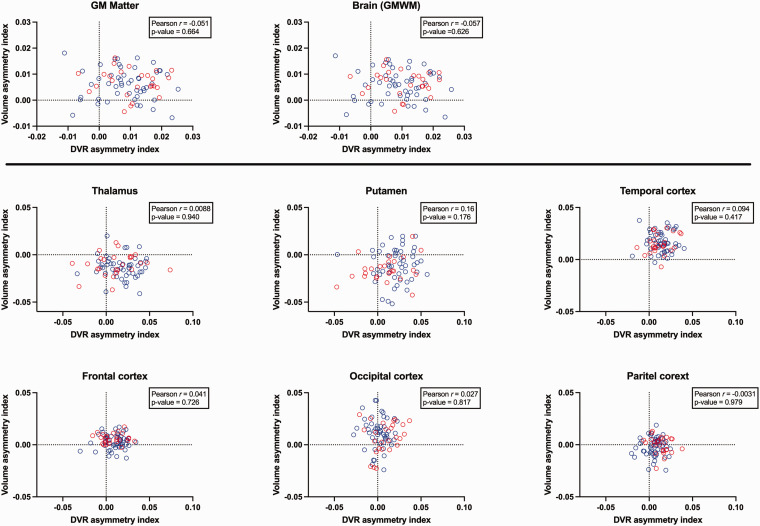
Pearson correlation between the volume asymmetry index and [^11^C](*R*)PK11195 DVR hemispheric asymmetry index at both global and regional levels. The text box within each figure reports the Pearson r-values and associated p-values. The dashed line represents the zero level, and a positive sign for the indices indicates that the right is greater/larger than the left.

Global volumetric analysis revealed significantly larger right hemisphere volumes compared to the left hemisphere for both grey matter and brain (GMWM) (p < 0.001). Regionally, findings varied: the left thalamus and putamen were significantly larger than their right counterparts (p < 0.001), while the right temporal, frontal, and occipital cortices were significantly larger than those on the left (p < 0.001). No significant volumetric asymmetry was observed between the left and right parietal cortices (p = 0.194). The direction of volumetric asymmetry can be visually confirmed in Figure 3. A positive sign indicates that the right hemisphere volume is greater than the left. Correlation analysis (also shown in Figure 3) revealed no significant relationships between volumetric and [^11^C](*R*)PK11195 DVR asymmetry indices, neither globally nor regionally. Notably, DVR asymmetry consistently was higher in the right side, even in brain regions with left structural asymmetries (e.g., the thalamus) or no volumetric asymmetry (e.g., parietal cortices).

Returning to Table 2, the [^11^C](*R*)PK11195 DVR for all six bilateral brain regions on the left and right sides originating from Manchester and Turku are presented. For within-centers evaluation of hemispheric differences, the results from Manchester dataset showed statistically significant higher DVR in the right regions than the left regions everywhere except the occipital cortex, where this difference did not reach significance (p = 0.237; Wilcoxon test). In the Turku dataset, all six brain regions showed significantly higher right than left DVR (p < 0.05; Wilcoxon test). The highest significance was reached with p < 0.001 in the parietal cortex and the temporal cortex (Table 2).

To allow for the possibility that the individual biological factors handedness (left/right), age, and sex (male/female) along with PET centre confounded the post-hoc analyses, a linear mix effect model incorporated these factors as fixed parameters in addition to the hemisphere (left/right GMWM DVR) was used (Table 3). The Shapiro-Wilk test indicated that the residuals of the statistical model were normally distributed. The estimated difference between the two hemispheres (right vs. left) is 0.020, meaning the right hemisphere showed higher DVR values, and the difference was statistically highly significant (p < 0.001). In contrast, the variables age, sex, handedness, and PET centre had no significant effect on DVR values (last column in Table 3).

**Table 3.** The relationships of hemisphere, age, sex, handedness and PET centre to the brain (GMWM) [^11^C](*R*)PK11195 DVR, N = 67/76 for the analysis.

Fixed effects	Estimate (standard deviation)	P value
Hemisphere (right vs. left)	0.020 (0.0026)	<0.001
Age	−0.00062 (0.00054)	0.253
Sex (male vs female)	−0.0079 (0.015)	0.600
handedness (right vs. left)	0.029 (0.022)	0.197
PET Centre (Turku vs. Manchester)	−0.012 (0.017)	0.499
Centre-by-hemisphere interaction	0.0010 (0.0050)	0.841

The regional fixed effect estimates with confidence intervals and p-values for the left and right difference in [^11^C](*R*)PK11195 DVR are presented in Table 4. Significant differences between right regions and left regions were observed in all regions, with right regions showing higher [^11^C](*R*)PK11195 DVR. For example, the right thalamus DVR was 0.041 higher than the left thalamus, and similarly, the right temporal cortex DVR values were 0.030 higher than the left temporal cortex.

**Table 4.** Fixed effects estimate with 95% confidence intervals and p-values for left and right as regressors for regional [^11^C](*R*)PK11195 DVR. N = 67/76 for the analysis.

Region	Estimate (group)	95% CI lower	95% CI higher	p-value
Thalamus (GMWM)	0.041	0.028	0.054	<0.001
Putamen (GMWM)	0.047	0.035	0.059	<0.001
Temporal Cortex	0.030	0.023	0.037	<0.001
Frontal Cortex	0.024	0.016	0.031	<0.001
Occipital Cortex	0.003	−0.005	0.010	<0.001
Parietal Cortex	0.012	0.006	0.019	<0.001

GMWM: grey matter and white matter tissues combined; CI: confidence intervals.

## Discussion

This study presents the largest [^11^C](*R*)PK11195 PET dataset of 76 healthy participants from two centres, aiming to elucidate asymmetry in TSPO expression in the normal brain. Results reveal that [^11^C](*R*)PK11195 shows global and regional asymmetry, with higher levels in the right brain regions. This asymmetry was independent of handedness and biological factors, including age and sex.

Few studies have separately examined left and right brain regions using TSPO PET imaging, and study design limitations may influence their findings. One key factor is sample size, as smaller samples reduce statistical power, making it harder to detect subtle hemispheric asymmetries. For example, Kumar et al.^46^ tested hemispheric asymmetry using [^11^C](*R*)PK11195 in 15 healthy adults and reported no significant difference between left and right brain regions, a result that may be attributable to their limited sample size. Additionally, Gershen et al.^29^ assessed hemispheric asymmetry in their clinical study using two second-generation TSPO radiotracers, [^11^C]PBR28 (11 controls) and [^11^C]DPA-713 (7 controls), and reported significant hemispheric asymmetry in the fusiform gyrus with [^11^C]DPA-713 but not with [^11^C]PBR28.^29^ Although the sample sizes were limited, these findings suggest that TSPO asymmetry may be radiotracer-specific, as different tracers have varying affinities for TSPO and are influenced by genetic factors affecting binding potential. However, discrepancies between studies may arise from differences in PET instrumentation and quantification methods, which could influence the sensitivity to detect asymmetries. Studies using lower-resolution PET scanners may be more prone to partial volume effects, which could obscure subtle differences in TSPO expression. Our study utilized the HRRT scanner, the highest-resolution brain scanner currently available. This increased resolution may enhance the detection of subtle hemispheric differences in TSPO expression that were previously undetectable. Therefore, the detection of TSPO asymmetry may depend on imaging methods, indicating the need for further research with larger samples and standardized protocols to improve understanding of hemispheric TSPO expression.

Although the underlying causes of [^11^C](*R*)PK11195 asymmetry are unclear, it is plausible that structural/anatomical brain asymmetry may play a role. Structural brain asymmetries are often associated with functional specialization; for example, regions involved in language processing, such as the planum temporale, are typically larger in the left hemisphere, whereas areas involved in spatial and creative tasks, like the anterior cingulate cortex, tend to be larger in the right hemisphere.^71^ Additionally, the brain cortical thickness, a measure of the thickness of the cerebral cortex,^72,73^ is larger in right hemisphere regions, including lateral and medial parts of the temporal, parietal, and occipital cortices.^74^ Similarly, the brain cortical area, a measure of the surface area of the cerebral cortex,^75,76^ has rightward asymmetry in the caudal anterior cingulate cortex and the middle temporal gyrus.^74^ We assessed volumetric asymmetry globally and regionally and examined its correlation with [^11^C](*R*)PK11195 DVR asymmetry. Although structural asymmetries were evident, they did not correlate significantly with DVR asymmetries at either global or regional levels. Thus, differences in brain structure do not appear to explain the observed TSPO asymmetry, suggesting that underlying physiological differences in binding affinity are likely responsible. In addition to structural considerations, differences in cellular density might also influence TSPO asymmetry, though this possibility was not directly tested here. Future studies focusing on cellular density, potentially using imaging techniques such as diffusion tensor imaging (DTI), which provides insights into tissue microstructure, could provide further insight into the underlying mechanisms driving these asymmetries.

The occipital cortex shows the lowest asymmetry (p = 0.012). This observation may reflect the inherent functional symmetry of the occipital cortex, which is primarily concerned with vision, a process that requires similar contributions from both occipital hemispheres.^77^ Thus, it is plausible that the relatively low TSPO asymmetry in this region reflects this symmetrical role. Primary visual cortex, in particular, has a highly symmetric function^78^ and therefore is not expected to have the same lateralisation as other brain regions, for example, controlling motor function.

Post-mortem TSPO studies^1,47–57^ have not reported left and right ROIs in the brain. This lack of data limits comparing post-mortem findings with our in vivo results. Post-mortem studies lack blood circulation, which may affect TSPO binding and brain functions, unlike PET studies. Blood circulation is closely tied to neuronal activity and TSPO expression in living brains, making direct comparisons difficult. Therefore, caution must be taken when interpreting post-mortem findings in the context of TSPO PET data.

Several PET studies have previously reported hemispheric asymmetry in cerebral glucose metabolism,^32–35^ with the right hemisphere showing more activity in the lateral frontal and temporal lobes and cerebellum, while the left showed more activity in the lingual gyrus, medial frontal gyrus, thalamus, and superior cingulate.^34^ Similarly, several PET and SPECT studies demonstrated hemispheric asymmetry in cerebral blood flow^36–41^ and consistently reported higher cerebral blood flow in the right hemisphere.^41^ Our findings add to this by demonstrating global and regional hemispheric asymmetry in [^11^C](*R*)PK11195 binding, with notably higher binding in the right brain regions. This finding suggests a potential link between TSPO expression and broader hemispheric asymmetry in immunological functions in healthy brains. Interestingly, our results mirror animal studies indicating a higher production of interleukins in the right hemisphere compared to the left.^7^ Despite these observations, direct evidence of asymmetrical immunological function in the human brain remains limited. Future studies should explore the mechanisms behind the hemispheric differences in TSPO expression and their relevance to immunological processes.

Understanding the normal distribution of TSPO in healthy brains is essential for distinguishing physiological variation from pathology-associated changes. Studies have evaluated TSPO expression using PET imaging in several diseases. For example, *Cagnin* et al.^9^ evaluated TSPO expression using [^11^C](*R*)PK11195 PET in Alzheimer’s disease (AD) patients versus healthy controls, and [^11^C](*R*)PK11195 binding was reported in left and right brain regions. The regions were manually defined on MRI and are not directly comparable with the atlas ROIs defined in this study. The same kinetic model was used (SRTM), but a different reference tissue input function (unsupervised cluster analysis versus cerebellar grey matter); therefore, the two studies have different absolute values of binding potential. In many regions, there is good agreement of the relative hemispheric difference in the control mean DVR; for example, the thalamus +2.8%^9^ versus +3.1% in this study. However, out of the 16 reported regions, three regions had higher binding in the left than the right hemisphere (fusiform gyrus, posterior cingulate gyrus, and putamen),^9^ which was not found in this study. The study also showed higher [^11^C]*(R)*PK11195 binding in AD patients than in the healthy controls across several brain regions. The most significant region listed was the inferior and middle temporal gyri, with a 10% higher mean DVR in the AD cohort than the controls.^9^ Compared to glucose metabolism PET study in AD patients, the measurements of cerebral metabolic rates of glucose (CMR_glc_) show lower values than in controls, with the biggest decrease seen in the left parietal cortex: ${\text{CMR}}_{\text{glc}}^{\text{normal}}$ = 7.77 ± 1.10 mg/100 g/min and ${\text{CMR}}_{\text{glc}}^{\text{AD}}$ = 5.27 ± 00 mg/100 g/min.^79^ This corresponds to a 32% reduction of CMR_glc_ in AD.^79^ This comparison emphasizes the implication of understanding the normal background [^11^C](*R*)PK11195 binding asymmetry, as there are subtle differences in binding between neuropathologic conditions and healthy controls.

The observed asymmetry has implications for the analysis and design of future TSPO PET studies. Physiological asymmetry in [^11^C](*R*)PK11195 binding is likely to affect how tracer uptake is interpreted in disease conditions. If the asymmetry is not considered, it may lead to incorrect estimates of pathology. The impact of the asymmetry of [^11^C](*R*)PK11195 binding in the normal brain is immediately clear in a study with a lateral lesion, for instance, a brain tumor or stroke. As an example, if a lesion was in the left frontal cortex and had a DVR of 1.12, then the DVR_lesion_ would be 2.00% higher relative to the bilateral frontal cortex DVR of 1.10, 3.29% higher relative to the median left frontal cortex DVR of 1.08 and 0.75% higher relative to the median right frontal cortex DVR of 1.11, respectively. The asymmetry effect in studies of non-localised brain disorders is more complex to predict. Simply averaging the values for a brain structure on the left and right side may not only be inaccurate in terms of comparing it with an averaged out left and right reference value in the normal brain that does not account for the hemispheric difference of [^11^C](*R*)PK11195 binding described in this study. It also entirely ignores the possibility that the brain disorder could have led to different changes in [^11^C](*R*)PK11195 binding in each hemisphere. We therefore strongly recommend to separately report the values for left and right structures in cross-sectional [^11^C](*R*)PK11195 studies, and then to conduct separate statistical tests for differences with hemisphere-specific reference values.

The results indicate handedness does not affect [^11^C](*R*)PK11195 asymmetry. This can be explained from a functional view, particularly in how TSPO imaging differs from, for example, glucose metabolism. The lack of a relationship between handedness and TSPO asymmetry arises from these biomarkers' distinct roles in brain function. Glucose metabolism PET imaging revealed handedness-related asymmetries in motor areas by highlighting differences in metabolic activity.^33^ Handedness is closely tied to lateralized brain functions, such as motor control and language processing,^31^ which are mainly influenced by neuronal activity and metabolism, not inflammation or microglial baseline expression. TSPO imaging, on the other hand, mainly reflects cellular and microglial states that may not be directly associated with the lateralized brain functions that underlie handedness. Given that handedness is a characteristic of neuronal connections and functional specialization, its connection to glial activity or TSPO asymmetry would be minimal in a healthy brain. Without clear pathology, TSPO asymmetry might indicate normal differences in glial activity instead of functional variations.

In this study, the results indicate age and sex do not significantly affect [^11^C](*R*)PK11195 binding. TSPO expression and its association with age and sex have been evaluated previously by [^11^C](*R*)PK11195 PET and other TSPO PET radiotracers.^9,20,46,80–86^ Reports were inconclusive regarding the extent of the relationship between age and TSPO PET measures. Some studies^80,81,83^ reported no change in TSPO binding related to age, which is consistent with our results. Other studies^46,82,86^ reported widespread positive increases in TSPO binding in some brain regions with age, and a study reported that TSPO binding only increases with age in the frontal and temporal cortex.^85^ Quantification approaches with different reference tissue input functions affect the results regarding age-related effects on TSPO binding.^87^ Therefore, a full quantification approach using plasma input function is necessary to accurately assess age-related TSPO binding.

Although newer second-generation TSPO radioligands with improved signal-to-noise ratio have been developed and introduced into clinical research, this study still employed the prototypical TSPO radiotracer [^11^C](*R*)PK11195 in use for over forty years. First, it remains the most widely used TSPO ligand in clinical PET studies. According to a review by Chauveau et al. (2021),^42^ out of 3914 patients who underwent a TSPO PET scan, 1851 patients (47%) received [^11^C](*R*)PK11195 with [^11^C]PBR28 the second most used tracer (938 patients or 24%). However, accurate quantification of [^11^C]PBR28 brain binding uses individually acquired blood data for the generation of a plasma input function whereas reference tissue quantification approaches have been established for [^11^C](*R*)PK11195 brain scans^87^ making the PET imaging procedure tolerable in vulnerable study populations which was the second reason for us to choose [^11^C](*R*)PK11195 in this investigation. Finally, the prototypic tracer [^11^C](*R*)PK11195 is indifferent to the allelic status reflecting a functional polymorphism of the TSPO gene.^88^ Therefore also, low-affinity binders can be included in [^11^C](*R*)PK11195 imaging studies. Whilst we acknowledge the advantages of higher specific binding of newer TSPO radiotracers for quantitative PET imaging, [^11^C](*R*)PK11195 continues to be used and therefore describing its hemispheric binding asymmetry remains relevant for clinical research and provides the starting point for investigating the *in vivo* properties of recently developed TSPO tracers in the human brain.

An assumption on the observed asymmetries obtained in [^11^C](*R*)PK11195 binding in the normal brain between the left and right hemispheres part of the brain could be a consequence of PET camera sensitivity imperfections. However, this is highly unlikely due to the meticulous maintenance of the HRRT scanners used for this study. These scanners undergo periodic normalization scans to account for detector blocks that might present some reduction of sensitivity as part of the aging of the detectors or set-up imperfections in the camera. These normalization scans, a standard procedure in PET cameras, measure differences in individual crystal efficiency, which, in the case of the HRRT, is performed with a germanium-69 rod source to obtain correction factors, which are later applied during reconstruction to the acquired emission data. In addition, regular quality controls using a cylindrical filled phantom with Germanium-68 and fluorine-18 (for dose calibration cross-calibration) were also conducted while the HRRT scanner was operational. The image uniformity was assessed periodically quantitatively and qualitatively during those checks to evaluate any issues with the camera setup. Furthermore, the data presented in this study are from two independent PET research sites, further minimizing the possibility of artificial asymmetry due to PET camera detectors' insensitivity. Another consideration is the potential minor systematic laterality effects introduced during processing and co-registration. The DVR difference (DVR_right_–DVR_left_) exhibits a Gaussian distribution, typical of biological factors and not from systematic technical errors.

Other sources of radiotracer uptake asymmetries could result from scattering outside the FOV, such as heart scattering on the reconstructed PET brain images outside the field of view (FOV). This is particularly relevant in our study because the heart is typically on the left side of the body and is one of the tissues with the highest TSPO expression.^89^ However, HRRT PET systems are equipped with dedicated tungsten shielding, a feature that significantly reduces the scanner's sensitivity to activity outside the FOV,^59^ and one can hypothesize that the effects would be distributed randomly between the left and right brain structures rather than consistently displaying one-sided asymmetry.

Lastly, when combining already collected datasets across different studies and centres, data collection protocols are not harmonized prospectively, which might increase data heterogeneity. However, this heterogeneity can be taken as an advantage of our approach in the sense that our findings are not limited to a single centre. We also statistically control for the possible centre differences by treating the PET centre as a fixed effect and including a centre-by-hemisphere interaction term. Neither the estimated effect of the PET centre nor the interaction between centre and hemisphere were statistically significant, suggesting that the observed asymmetries in [^11^C](*R*)PK11195 binding are more likely related to variations in physiological uptake rather than systematic differences between centres.

Our study demonstrates a global and regional asymmetry in [^11^C](*R*)PK11195 binding, with significantly higher uptake in the right hemisphere across multiple brain regions. The asymmetry is independent of handedness, age, and sex. Understanding this normal binding asymmetry is crucial for accurately discriminating between normal TSPO expression and pathological alterations in clinical settings.
